# Longitudinal assessment of the inflammatory response: The next step in personalized medicine after severe trauma

**DOI:** 10.3389/fmed.2022.983259

**Published:** 2022-09-20

**Authors:** E. J. de Fraiture, N. Vrisekoop, L. P. H. Leenen, K. J. P. van Wessem, L. Koenderman, F. Hietbrink

**Affiliations:** ^1^Department of Trauma Surgery, University Medical Center Utrecht, Utrecht, Netherlands; ^2^Department of Surgery, Sint Antonius Hospital, Nieuwegein, Netherlands; ^3^Department of Respiratory Medicine, University Medical Center Utrecht, Utrecht, Netherlands; ^4^Center for Translational Immunology (CTI), University Medical Center Utrecht, Utrecht, Netherlands

**Keywords:** trauma, inflammatory, neutrophil, infection, longitudinal

## Abstract

Infections in trauma patients are an increasing and substantial cause of morbidity, contributing to a mortality rate of 5–8% after trauma. With increased early survival rates, up to 30–50% of multitrauma patients develop an infectious complication. Trauma leads to a complex inflammatory cascade, in which neutrophils play a key role. Understanding the functions and characteristics of these cells is important for the understanding of their involvement in the development of infectious complications. Recently, analysis of neutrophil phenotype and function as complex biomarkers, has become accessible for point-of-care decision making after trauma. There is an intriguing relation between the neutrophil functional phenotype on admission, and the clinical course (e.g., infectious complications) of trauma patients. Potential neutrophil based cellular diagnostics include subsets based on neutrophil receptor expression, responsiveness of neutrophils to formyl-peptides and FcγRI (CD64) expression representing the infectious state of a patient. It is now possible to recognize patients at risk for infectious complications when presented at the trauma bay. These patients display increased numbers of neutrophil subsets, decreased responsiveness to fMLF and/or increased CD64 expression. The next step is to measure these biomarkers over time in trauma patients at risk for infectious complications, to guide decision making regarding timing and extent of surgery and administration of (preventive) antibiotics.

## Outline

Trauma leads to a complex inflammatory cascade that can cause an acquired immunodeficiency, which makes patients prone to develop infectious complications (1). Neutrophils play a key role in these processes (2). Distinct neutrophil subtypes with different functions exist, and these cells participate in the development and/or recovery of infectious complications after trauma (3–5). Recently, analysis of neutrophil phenotype and function as complex biomarkers has become accessible for point-of-care decision-making in the management of trauma patients (6). The aim of this narrative review is to discuss the potential use of longitudinal assessment of the neutrophil compartment by point-of-care flow cytometry. The goal of these sequential measurements over time is to guide clinical decision-making regarding end goals of resuscitation, indication and timing of immune protective surgery in a damage control setting as well as definitive surgery and the administration of (preventive) antibiotics.

The immunological response to trauma is initiated at the moment of injury. At this point, two inflammatory responses are induced in parallel. The systemic pro-inflammatory response to tissue damage makes patients susceptible to multi organ dysfunction syndrome (MODS), while the simultaneous immune paralysis makes patients susceptible to late infectious complications (7). Additional trauma caused by (operative) interventions can worsen this inflammatory response, thereby, increasing the risk of inflammatory and infectious complications (1). From a clinical perspective, these processes can complicate the course of trauma patients by early pro-inflammatory complications during the first 4 days after trauma and by late-onset (>5 days) sepsis, which is suggested to have a relation with the magnitude of the inflammatory response in the beginning (8–11).

This relation between extent of the inflammatory response and the activation of the immune system can be measured by multiple neutrophil biomarkers for both phenotype and function (12). The next step is to measure neutrophil biomarkers at different time points after trauma to guide decision making regarding timing and burden (i.e., approach and extent) of surgery and administration of (preventive) antibiotics.

## Clinical relevance

Injury is the leading cause of death in young individuals (13). Due to advances in the organization of trauma care, hemorrhage control, surgical approaches and resuscitation, overall mortality after trauma has declined up to 75% in recent decades (14–17). With increased early survival rates, infections are the most common complications affecting trauma patients (18, 19). Thus, infections are an increasing and substantial cause of morbidity, contributing to a mortality rate of 5–8% after trauma (17, 20). A large number of trauma patients are able to survive the first critical phase after trauma, which creates new challenges in terms of diagnostics and treatment in longitudinal management of trauma patients.

Injury induces an immediate innate immune response, leading to a complex inflammatory cascade that induces both immune activation and a refractory immune state in parallel (21). Neutrophils are the first immune cells responding to tissue damage and invading pathogens and they are known to play a key role in regulating the inflammatory response after trauma (2). Recently, analysis of neutrophil phenotype and function (e.g., neutrophil size, neutrophil subsets, neutrophil activation markers, responsiveness of these markers to bacterial stimuli) has become accessible to such extent that they can be used for point-of-care (POC) decision making after trauma (6).

Until now, studies mainly focused on the assessment of the neutrophil functional phenotype on admission at the trauma bay. In this setting, a relation between neutrophil phenotype on admission and the clinical course of trauma patients (e.g., inflammatory, and infectious complications) was demonstrated (8–11). However, the course of the inflammatory response is dynamic in time and is driven by both patient characteristics, injury type and severity, timing and burden (i.e., extent) of (operative) interventions. Further determination of these changes over time will increasingly facilitate personalized care of injured patients. As it is now possible to measure the neutrophil functional phenotype at any time point after trauma in a fast and non-labor intensive manner (12), the longitudinal course of the inflammatory response and the impact of additional interventions can be measured.

## Inflammatory responses after trauma

The innate immune response is initiated by injury-related tissue damage, coined as “the first hit” (22). The amplitude of this response is thought to be determined by the severity and type of injury (6, 9, 11, 23). The response is characterized by two responses developing in parallel. Firstly, a **pro-inflammatory** **response** is mediated by a systemic inflammatory response to tissue damage, in reaction to damage-associated molecular patterns (DAMPs) and microbe-associated molecular patterns (MAMPs) (24). A dysregulated pro-inflammatory response can increase susceptibility to (multi) organ failure such as ARDS or MODS (9). Neutrophils might also help to resolve tissue damage by the release DNA structures that are formed like a web, namely neutrophil extracellular traps (NETs). NETs could contribute to preventing dissemination of (larger) pathogens (25). However, when neutrophils produce too many NETs as a reaction to trauma, these toxic structures further amplify the pro-inflammatory response, contributing to an imbalanced immune reaction (26). Secondly, an **anti-inflammatory** **response** is mediated by a refractory immune state, which leads to immune paralysis and susceptibility to infectious complications e.g., sepsis. Recently, it became clear that although these responses appear clinically distinct, the underlying immunological mechanisms, in terms of the appearance of neutrophil subsets and pro-inflammatory cytokines e.g., IL-6 and IL-10, start simultaneously and immediately after the injury (8, 27–30).

Additional trauma caused by surgical intervention, generally referred to as “a second hit,” can contribute to a further dysregulation of the immune response, thereby increasing the risk of inflammatory/infectious complications (31). Over the past decade surgical procedures were shortened to minimize the effect of such a second hit. This physiological and immune protective surgery is becoming more and more recognized as important in the early phases of treatment after trauma. Over time, different strategies have been practiced for treating multitrauma patients that are considered too unstable for early total care. The concept of damage control surgery (DCS) has come up since the 1990s, focusing on improving survival by rapidly controlling hemorrhage and contamination by a brief initial operation. Only when a patient becomes hemodynamically stable, definitive surgery is performed (22). Nowadays, a strategy that focusses on early fixation of bones, combined with a continual reassessment of the reaction and response to injury and surgery, has gained popularity. When physiology (e.g., in terms of physiological or immunological response) deteriorates, fixation should be delayed until the patient stabilizes (32). This strategy is coined early appropriate care.

Over the past decades, due to advanced hemorrhage control (damage control surgery and damage control orthopedics) and (damage control) resuscitation, a decrease in mortality and a significant lowering of the incidence of inflammatory complications (e.g., ARDS, MODS) was observed (33–35). This had consequences for studies before 2010 and after 2012, during which the inflammatory marker interleukin-6 (IL-6) was measured (11, 36, 37). In the earlier period 2006–2010 higher injury severity scores (ISS) and higher concentrations of IL-6 were found to correlate with inflammatory complications. Interestingly, this correlation between IL-6 and ISS was lost in studies published after 2012, where a lower incidence of inflammatory complications was observed (see Table 1) (11, 36–38). Thus, despite a pronounced initial inflammatory response, the systemic inflammation was lower leading to a decrease in inflammatory complications most likely due to ongoing better management of trauma care (39).

**TABLE 1:** Incidence of SIRS, sepsis and ARDS were tested using Fisher’s exact test.

	Early period	Late period	*P*-value
Number of patients (n)	19	15	
Study period	2006–2010	2012–2016	
SIRS	14	14	0.18
Sepsis	4	1	0.34
ARDS/MODS	8	0	0.003*

Significant value in indicated with *. SIRS, systemic inflammatory response syndrome; ARDS, acute respiratory distress syndrome; MODS, multiple organ dysfunction syndrome.

In addition, in a cohort study with thirty-three patients with acute burn patients, a clear dose response relation was found between the degree of skin injury and the percentage of banded neutrophils. Furthermore, patients with additional thermal inhalation injuries were characterized by a decreased responsiveness of fNLF of neutrophils in peripheral blood. This indicates that burn injuries cause a mild activation of systemic cellular inflammation, and that additional thermal inhalation injury induces refractory systemic neutrophils, possibly leading to an increased susceptibility to infectious complications. These results are not published.

Currently, the mortality after trauma is increasingly caused by neurotrauma (33, 40, 41). This illustrates that the pro-inflammatory response, that used to be causing hyper-inflammatory systemic conditions, seems more under control (33). This leads now to an anti-inflammatory imbalance with new clinical challenges, such as recognition and treatment of late (>5 days) infections mainly resulting in increased morbidity. A dysregulation of the immune response, in which neutrophils play a pivotal role, predisposes patients to these infectious complications (11). Understanding the functions and characteristics of neutrophils is important to understand their involvement in the development of infectious complications.

## Potential neutrophil biomarkers for treatment of trauma patients

The immediate immune response directly after trauma is regulated by many humoral and cellular mediators including leukocytes, coagulation, systemic cytokine release and complement cascades (42). Neutrophils are the most abundant innate effector cells and belong to a final common pathway causing tissue damage upon hyper activation of these cells (2). Therefore, research over the past decades has focused on identification of complex activation of neutrophils to identify trauma patients with high risk on adverse outcome. An overview of the investigated markers is described below.

### Cell size and granularity of neutrophils

White blood cell count (WBC) and differentiation into leukocyte subsets have been extensively investigated in trauma patients. A marked increase of WBC and especially neutrophilic granulocytes has been noted after trauma, but this increase in cell numbers *per se* was found to be of no prognostic value (43, 44). In addition, WBC morphological parameters have been assessed to identify patients at risk. An increase in neutrophil cell size was observed in non-survivors after severe trauma, implying an increased cell size to be a predictor for mortality (45). Thereafter, in another cohort, a rise in neutrophil cell size preceded the clinical manifestation of organ dysfunction in every patient with a complication (46). Unfortunately, it remains to be determined which precise mechanisms underlie the increase in neutrophil cell size.

A partial explanation for an increased cell-size could be the influx of immature neutrophils in the peripheral blood (47, 48). It is known that immature neutrophils appear in the bloodstream as a result of the inflammatory reaction immediately after trauma; generally referred to as a left shift (49, 50). These young neutrophils with a larger cell size are known to have a C shaped (“band”) nucleus instead of a lobulated nucleus. The neutrophil left shifts have proven their diagnostic value in patients with a bacterial infection (51). However, routine analyzers are not able to recognize a left shift of banded neutrophils in trauma patients, while they are able to do so in infectious patient groups (52). So, the question remains if banded neutrophils in trauma patients are different in function and phenotype from banded neutrophils in patients suffering from an infection. Further research has focused on phenotyping neutrophils based on receptor expression (CD16/CD62L), as this is a more accurate method to identify different phenotypes of neutrophils in trauma patients (6).

### Subsets of neutrophils

**FIGURE 1:**
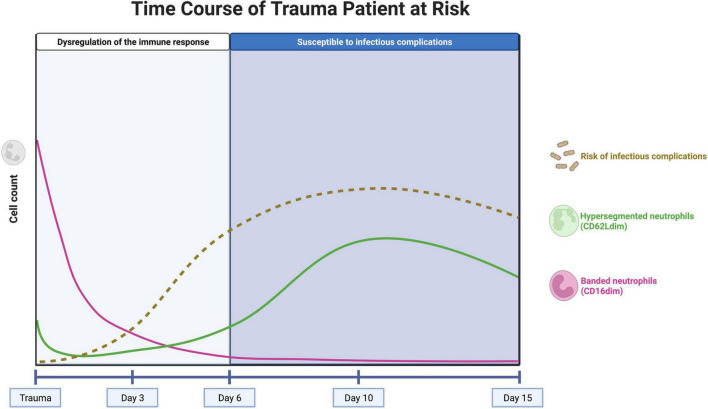
Concept of the neutrophil subset response over time in a trauma patient at risk for infectious complications. The pink line represents the course of well-functioning banded (CD16^dim^) neutrophils, entering the circulation immediately after trauma. The influx of these cells stops after approximately 1 day and the neutrophils have an average lifetime of 5 days. The green line represents the course of hypersegmented (CD62L^dim^) neutrophils, with impaired killing capacity. These cells appear after several days, with a peak after 10 days. The brown, dashed line represents the risk of infectious complications over time. The risk increases after 3–5 days, when there are no banded neutrophils left in the circulation. After 10 days, the peak of hypersegmented neutrophils coincides with the first systemic infectious complications. Created with BioRender.com.

Under homeostatic conditions, only a homogeneous population of mature neutrophils identified by CD16^bright^/CD62L^bright^ cells circulate in the peripheral blood. However, during acute inflammation, neutrophils in the peripheral blood can be divided into different subsets based on the expression of specific surface proteins (CD16/FcγRIII and CD62L/L-selectin) (3). Immediately after trauma, large numbers of immature neutrophils with a banded shaped nucleus (CD16^dim^/CD62L^bright^) enter the circulation and after several days also hypersegmented neutrophils (CD16^bright^/CD62L^dim^) can be observed (Figure 1) (50, 53). Interestingly, these neutrophil subsets based on the expression of CD16 and CD62L have different functions (3, 54). Landmark studies in healthy controls, who received lipopolysaccharide (LPS) inducing systemic inflammation, showed that banded (immature) neutrophils display a higher killing capacity than segmented neutrophils and that hypersegmented neutrophils are associated with almost immediate bacterial outgrowth, due to impaired intracellular killing after adequate phagocytosis (3). In addition, polytrauma patients who developed infectious complications had significantly higher percentage CD16^dim^/CD62L^bright^ (banded) neutrophils in the blood immediately after trauma compared with polytrauma patients who did not develop infectious complications (6).

Neutrophil left shifts (influx of immature CD16^dim^ (banded) neutrophils into the blood) are observed immediately after trauma but quickly disappear after approximately 1 day (5). This well-known phenomenon is explained by a massive mobilization of neutrophils from the bone marrow (50). This can lead to a subsequent imbalance of the neutrophil compartment and the bone marrow might be unable to fully compensate in time for the loss of these well-functioning neutrophils that can live up to 5 days under homeostatic conditions (55, 56). This phenomenon might be the driving force for the clinical observation that the trauma patient becomes highly sensitive to infectious complications after 5–6 days post trauma (Figure 1) (57).

On the other hand, CD62L^dim^ (hypersegmented) neutrophils appear in the peripheral blood several days (>3 days) after trauma with a peak after 10 days (5). These CD62L^dim^ neutrophils were shown to have impaired killing capacity and a reduced acidification capacity, but they have immunoregulatory characteristics that could be important for keeping the balance in the immune response (54). The presence of high numbers of CD62L^dim^ neutrophils during the days to weeks following severe trauma might play a significant role in the risk of infectious complications in trauma patients but this concept needs more experimental support. It is, however, known that the first systemic infectious complications that have consequences for the clinical course of the patient arise at the end of the first week after trauma (58). This coincides with the rising counts of CD62L^low^ cells during the first week after trauma, so it is tempting to hypothesize that high numbers of CD62L^low^ neutrophils can lead to an increased risk of infectious complications (Figure 1) (10).

Trauma patients with high numbers of banded neutrophils directly after trauma and high numbers of hypersegmented neutrophils after 10 days in the peripheral blood seem also at risk for infectious complications (Figure 1). In these patients, longitudinal measurement of the neutrophil compartment and the amount of CD16^dim^ and CD62L^dim^ neutrophils could guide the clinician regarding decisions on the timing and extent (burden) of surgery. A patient that is not at risk could benefit from early total care, while a patient displaying extensive neutrophil subsets could benefit from damage control immune protective surgery.

### Responsiveness to inflammatory mediators (e.g., formyl-peptides/fMLF)

In addition to the assessment of neutrophil subsets by receptor expression, the functionality of neutrophils can be investigated in terms of responsiveness of neutrophil to formyl peptides (e.g., fMLF). fMLF activates neutrophils by interactions with their formyl peptide receptors (FPR) (59–63). A decreased responsiveness of neutrophils to fMLF at initial presentation was demonstrated in trauma patients who later developed septic shock (10). In addition, low responsiveness of neutrophils to fMLF immediately after trauma preceded by almost a week the development of septic complications after > 5 days (11). Thus, responsiveness to formyl-peptides appears to be a sensitive marker as it is involved in an essential part of the immune system that is activated during the pathogenesis of sepsis and septic shock. Modulation of this responsiveness can aid the clinician in identifying patients at risk. No longitudinal data has been published yet about the responsiveness of neutrophils in trauma patients. These patients might also benefit from longitudinal monitoring to guide decision making.

### Expression of FCγRI (CD64) on neutrophils

Specific expression of neutrophil receptors could be used as an extra tool to measure markers associated with specific components of the immune response. Neutrophil CD64 is a receptor that is upregulated on neutrophils within 1–6 h after administration of pro-inflammatory cytokines or bacterial cell wall components such as LPS (64). In the absence of a pro-inflammatory stimulus, CD64 levels on neutrophils start to decrease after 48 h and are return to baseline within 7 days (65). Thus, neutrophil CD64 is a relatively specific marker for bacterial infections (66). This property of CD64 has been utilized as a diagnostic marker of infection particularly for sepsis in intensive care patients (67). This could potentially also be a marker to aid the decision to early antibiotics in patients at risk for bacterial infections. Longitudinal measurement of CD64 expression in patients admitted at the hospital could aid the clinician with early diagnosis and follow up of bacterial infections (64, 68–71).

## Burden of surgery: Timing + intensity

**FIGURE 2:**
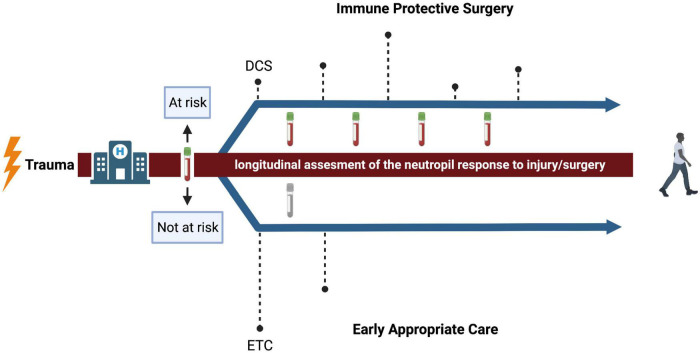
Conceptual representation of surgical strategies after trauma. The dashed lines represent the timing of surgical interventions and the height of the dashed lines represent the intensity. The upper arrow represents a proposed strategy for patients that, after flow cytometric measurement of the neutrophil compartment, are expected to be at risk for infectious complications. The impact of surgery is minimized and adjusted over time to reassessment of the neutrophil response. The lower arrow represents a proposed strategy for patients that are expected to be not at risk. Here, the approach of early appropriate care is suggested. DCS, damage control surgery; ETC, early total care. Created with BioRender.com.

There are different strategies for treating severely injured trauma patients. Damage control surgery (DCS) is characterized by a brief initial operation to stabilize the patient, followed by several short interventions (Figure 2) (22). This approach of physiological and immune protective surgery is commonly chosen when a patient is too unstable to survive definitive fixation in the acute moment (1). The alternative to damage control surgery is early total care (ETC) with definitive fixation within the first 24–36 h (Figure 2) (32). Recently, there has been a trend toward early appropriate care (EAC), which means providing a plan of action based on continuous assessment and reaction to the response to injury and surgery (31, 72). It is based on fixation of the fractured bones at an early stage, unless physiology deteriorates and definitive fixation should be postponed (32). However, the challenge is to correctly assess the response to injury and surgery to select the patients that benefit from delayed definitive fixation of bones.

Currently, there is no adequate method to estimate the physiological status of an injured patient over time. As shown in Figure 1, the trauma patient at risk of infectious complications is characterized by the presence of prominent neutrophil subsets in the peripheral blood directly after trauma (6). The change in the occurrence of these subsets over time might be associated with the risk of developing infectious complications (10). The impact of extensive surgery on the amount of neutrophil subsets in the blood and neutrophil responsiveness, has not yet been described. The next essential step is to investigate if the imbalance in the immune response further deteriorates, in terms of e.g., influx of banded/hypersegmented neutrophils of a further decreased responsiveness to fMLF, due to (extensive) surgery. Possibly, this could possibly aid decision making regarding timing and extent of surgery. Assessment of neutrophil subsets and responsiveness before and after surgery in patients that are deemed “at risk” directly after trauma is likely to give information about the impact of surgery and might give the opportunity to compare different surgical strategies. Thus, longitudinal assessment of changes in the neutrophil compartment might be the promising next step in personalized medicine in trauma patients, because it quantifies the inflammatory response caused by the first hit and subsequent hits.

## Antibiotic interventions

Infectious complications in trauma patients are treated with antibiotics. However, not all antibiotics may be useful at every moment after trauma. Patients with increased CD62L^dim^ subsets and decreased fMLF responses, could suffer from invading bacteria that reside inside the neutrophils for up to 5 days after trauma (3, 55, 73, 74). *Staphylococcus aureus (S. aureus*) is a common causative pathogen of infections after trauma and it is known that it has the ability to survive and proliferate inside neutrophils, as in a Trojan horse (3, 75). Treatment with antibiotics that could kill bacteria that are contained inside the neutrophil would protect vulnerable patients against severe infections that typically occur >5 days after trauma. The antibiotics quinolones, rifamycins and sulfamethoxazole-trimethoprim seem effective against *S. Aureus* inside a human neutrophil (74). Moreover, the infectious state of a patient should be monitored by measuring the neutrophil receptor CD64. In patients with a suspected infection, high levels of CD64 are of diagnostic value (66). Moreover, when the infection is diagnosed and antibiotics are administered, sequential measurement of CD64 gives early information on the effect of the antibiotics (76). If the expression of CD64 remains high, the bacteria could be resistant, or the antibiotic is not able to reach the bacteria. Later, a low expression of CD64 after cessation of antibiotic treatment, confirms a successfully treated infection. Thus, sequentially analyzing neutrophil subsets, estimating the killing capacity and monitoring the infectious status by CD64 on neutrophils may individualize both indication, timing and type of antibiotic used in severely injured trauma patients.

## Conclusion

The extent of the inflammatory response after trauma can be measured with an automatic point-of-care flow cytometry approach. It is known that there is a relation between the neutrophil functional phenotype on admission, and the clinical course (e.g., infectious complications) of the trauma patient. Potential neutrophil associated biomarkers include determination of subsets based on neutrophil receptor expression, responsiveness of neutrophils to formyl-peptides (fMLF) and CD64 expression representing the infectious state of a patient. By this technology it is likely possible to identify patients at risk for infectious complications when presented at the trauma bay. These patients display large extents of banded neutrophils directly after trauma and high numbers of hypersegmented neutrophils after 10 days, decreased responsiveness to fMLF and/or increased CD64 expression. The next step is to measure these biomarkers over time to guide decision making regarding timing and extent of surgery to achieve early appropriate care and determine the administration of (preventive) antibiotics.

## Author contributions

EF wrote the first draft of the manuscript. LK, LL, NV, KW, and FH critically reviewed the manuscript. All authors contributed to the article and approved the submitted version.

## References

[1] Lord JM Midwinter MJ Chen YF Belli A Brohi K Kovacs EJ The systemic immune response to trauma: an overview of pathophysiology and treatment. *Lancet.* (2014) 384:1455–65. 10.1016/S0140-6736(14)60687-5 25390327 PMC4729362

[2] Kubes P. The enigmatic neutrophil: what we do not know. *Cell Tissue Res.* (2018) 371:399–406. 10.1007/s00441-018-2790-5 29404726

[3] Leliefeld PHC Pillay J Vrisekoop N Heeres M Tak T Kox M Differential antibacterial control by neutrophil subsets. *Blood Adv.* (2018) 2:1344–55. 10.1182/bloodadvances.2017015578 29895625 PMC5998927

[4] Pillay J Ramakers BP Kamp VM Loi ALT Lam SW Hietbrink F Functional heterogeneity and differential priming of circulating neutrophils in human experimental endotoxemia. *J Leukoc Biol.* (2010) 88:211–20. 10.1189/jlb.1209793 20400675

[5] Bongers SH Chen N van Grinsven E van Staveren S Hassani M Spijkerman R Kinetics of neutrophil subsets in acute, subacute, and chronic inflammation. *Front Immunol.* (2021) 12:674079. 10.3389/fimmu.2021.674079 34248955 PMC8265311

[6] Spijkerman R Hesselink L Bongers S van Wessem KJP Vrisekoop N Hietbrink F Point-of-care analysis of neutrophil phenotypes: a first step toward immuno-based precision medicine in the trauma ICU. *Crit Care Explor.* (2020) 2:e0158. 10.1097/cce.0000000000000158 32766555 PMC7371075

[7] Hesselink L Spijkerman R Van Wessem KJP Koenderman L Leenen LPH Huber-Lang M Neutrophil heterogeneity and its role in infectious complications after severe trauma. *World J Emerg Surg.* (2019) 14:1–11. 10.1186/s13017-019-0244-3 31164913 PMC6542247

[8] Hietbrink F Oudijk EJ Braams R Koenderman L Leenen L. Aberrant regulation of polymorphonuclear phagocyte responsiveness in multitrauma patients. *Shock.* (2006) 26:558–64. 10.1097/01.shk.0000233196.40989.78 17117129

[9] Hietbrink F Koenderman L Althuizen M Leenen LPH. Modulation of the innate immune response after trauma visualised by a change in functional PMN phenotype. *Injury.* (2009) 40:851–5. 10.1016/j.injury.2008.11.002 19339006

[10] Hietbrink F Koenderman L Althuizen M Pillay J Kamp V Leenen LPH. Kinetics of the innate immune response after trauma: implications for the development of late onset sepsis. *Shock.* (2013) 40:21–7. 10.1097/SHK.0b013e318295a40a 23603769

[11] Groeneveld KM Koenderman L Warren BL Jol S Leenen LPH Hietbrink F. Early decreased neutrophil responsiveness is related to late onset sepsis in multitrauma patients: an international cohort study. *PLoS One.* (2017) 12:e0180145. 10.1371/journal.pone.0180145 28665985 PMC5493351

[12] Spijkerman R Hesselink L Hellebrekers P Vrisekoop N Hietbrink F Leenen LPH Automated flow cytometry enables high performance point-of-care analysis of leukocyte phenotypes. *J Immunol Methods.* (2019) 474:112646. 10.1016/j.jim.2019.112646 31419409

[13] CDC. *Top Ten Leading Causes of Death in the U.S. for Ages 1-44 from 1981-2020.* Atlanta, GA: CDC (2020).

[14] Murray CJL Lopez AD. Global mortality, disability, and the contribution of risk factors: global burden of disease study. *Lancet.* (1997) 349:1436–42. 10.1016/S0140-6736(96)07495-8 9164317

[15] GBD 2017 DALYS and Hale Collaborators. The global burden of disease. *Lancet.* (2018) 392:1859–922. 30415748 10.1016/S0140-6736(18)32335-3 PMC6252083

[16] World Health Organization. *The Injury Chart Book.* Geneva: World Health Organization (2002).

[17] Haagsma JA Graetz N Bolliger I Naghavi M Higashi H Mullany EC The global burden of injury: incidence, mortality, disability-adjusted life years and time trends from the global burden of disease study 2013. *Inj Prev.* (2016) 22:3–18. 10.1136/injuryprev-2015-041616 26635210 PMC4752630

[18] Komori A Iriyama H Kainoh T Aoki M Naito T Abe T. The impact of infection complications after trauma differs according to trauma severity. *Sci Rep.* (2021) 11:1–8. 10.1038/s41598-021-93314-5 34226621 PMC8257796

[19] Eguia E Bunn C Kulshrestha S Markossian T Durazo-Arvizu R Baker MS Trends, cost, and mortality from sepsis after trauma in the United States: an evaluation of the national inpatient sample of hospitalizations, 2012–2016. *Crit Care Med.* (2020) 48:1296–303. 10.1097/ccm.0000000000004451 32590387 PMC7872079

[20] Wafaisade A Lefering R Bouillon B Sakka SG Thamm OC Paffrath T Epidemiology and risk factors of sepsis after multiple trauma: an analysis of 29,829 patients from the trauma registry of the German society for trauma surgery. *Crit Care Med.* (2011) 39:621–8. 10.1097/CCM.0b013e318206d3df 21242798

[21] Hietbrink F Koenderman L Rijkers GT Leenen LPH. Trauma: the role of the innate immune system. *World J Emerg Surg.* (2006) 1:1–11. 10.1186/1749-7922-1-15 16759367 PMC1481567

[22] Rotondo MF Schwab CW McGonigal MD Phillips GR Fruchterman TM Kauder DR ‘Damage control’: an approach for improved survival in exsanguinating penetrating abdominal injury. *J Trauma Inj Infect Crit Care.* (1993) 35:375–83. 10.1097/00005373-199309000-00008 8371295

[23] Visser T Pillay J Pickkers P Leenen LPH Koenderman L. Homology in systemic neutrophil response induced by human experimental endotoxemia and by trauma. *Shock.* (2012) 37:145–51. 10.1097/SHK.0b013e31823f14a4 22089204

[24] Hazeldine J Hampson P Lord JM. The impact of trauma on neutrophil function. *Injury.* (2014) 45:1824–33. 10.1016/j.injury.2014.06.021 25106876

[25] Hidalgo A Libby P Soehnlein O Aramburu I Papayannopoulos V Silvestre-Roig C. Neutrophil extracellular traps.: from physiology to pathology. *Cardiovasc Res.* (2021) cvab329. 10.1093/cvr/cvab329 [Epub ahead of print]. 34648022 PMC9586562

[26] Wang J. Neutrophils in tissue injury and repair. *Cell Tissue Res.* (2018) 371:531–9. 10.1007/s00441-017-2785-7 29383445 PMC5820392

[27] Visser T Pillay J Koenderman L Leenen LPH. Postinjury immune monitoring: can multiple organ failure be predicted? *Curr Opin Crit Care.* (2008) 14:666–72. 10.1097/MCC.0B013E3283196522 19005307

[28] Xiao W Mindrinos MN Seok J Cuschieri J Cuenca AG Gao H A genomic storm in critically injured humans. *J Exp Med.* (2011) 208:2581–90. 10.1084/JEM.20111354 22110166 PMC3244029

[29] Hazeldine J Naumann DN Toman E Davies D Bishop JRB Su Z Prehospital immune responses and development of multiple organ dysfunction syndrome following traumatic injury: a prospective cohort study. *PLoS Med.* (2017) 14:1–29. 10.1371/journal.pmed.1002338 28719602 PMC5515405

[30] Timmermans K Kox M Vaneker M van den Berg M John A van Laarhoven A Plasma levels of danger-associated molecular patterns are associated with immune suppression in trauma patients. *Intensive Care Med.* (2016) 42:551–61. 10.1007/S00134-015-4205-3 26912315 PMC5413532

[31] Pape HC Giannoudis P Krettek C. The timing of fracture treatment in polytrauma patients: relevance of damage control orthopedic surgery. *Am J Surg.* (2002) 183:622–9. 10.1016/S0002-9610(02)00865-6 12095590

[32] Berwin JT Pearce O Harries L Kelly M. Managing polytrauma patients. *Injury.* (2020) 51:2091–6. 10.1016/j.injury.2020.07.051 32758368

[33] Van Wessem KJP Leenen LPH. Reduction in mortality rates of postinjury multiple organ dysfunction syndrome: a shifting paradigm? A prospective population-based cohort study. *Shock.* (2018) 49:33–8. 10.1097/SHK.0000000000000938 28682941

[34] van Wessem KJP Leenen LPH. Incidence of acute respiratory distress syndrome and associated mortality in a polytrauma population. *Trauma Surg Acute Care Open.* (2018) 3:e000232. 10.1136/tsaco-2018-000232 30623025 PMC6307585

[35] Sauaia A Moore EE Johnson JL Chin TL Banerjee A Sperry JL Temporal trends of postinjury multiple-organ failure: still resource intensive, morbid, and lethal. *J Trauma Acute Care Surg.* (2014) 76:582–93. 10.1097/TA.0000000000000147 24553523 PMC4116088

[36] Hietbrink F Koenderman L Leenen LPH. Intramedullary nailing of the femur and the systemic activation of monocytes and neutrophils. *World J Emerg Surg.* (2011) 6:1–6. 10.1186/1749-7922-6-34 22040874 PMC3216875

[37] Hietbrink F Koenderman L Van Wessem KJP Leenen LPH. The Impact of intramedullary nailing of tibia fractures on the innate immune system. *Shock.* (2015) 44:209–14. 10.1097/SHK.0000000000000405 26009818

[38] Heeres M Visser T van Wessem KJP Koenderman AHL Strengers PFW Koenderman L The effect of C1-esterase inhibitor on systemic inflammation in trauma patients with a femur fracture – the CAESAR study: study protocol for a randomized controlled trial. *Trials.* (2011) 12:223. 10.1186/1745-6215-12-223 21988742 PMC3198691

[39] van Wessem KJP Leenen LPH Hietbrink F. Physiology dictated treatment after severe trauma: timing is everything. *Eur J Trauma Emerg Surg.* (2022) 10.1007/s00068-022-01916-z [Epub ahead of print]. 35218406 PMC9532323

[40] Evans JA Van Wessem KJP McDougall D Lee KA Lyons T Balogh ZJ. Epidemiology of traumatic deaths: comprehensive population-based assessment. *World J Surg.* (2010) 34:158–63. 10.1007/S00268-009-0266-1 19882185

[41] Pfeifer R Tarkin IS Rocos B Pape HC. Patterns of mortality and causes of death in polytrauma patients–has anything changed? *Injury.* (2009) 40:907–11. 10.1016/J.INJURY.2009.05.006 19540488

[42] Huber-Lang M Lambris JD Ward PA. Innate immune responses to trauma review-article. *Nat Immunol.* (2018) 19:327–41. 10.1038/s41590-018-0064-8 29507356 PMC6027646

[43] Chang DC Cornwell EE Phillips J Paradise J Campbell K. Early leukocytosis in trauma patients: what difference does it make? *Curr Surg.* (2003) 60:632–5. 10.1016/j.cursur.2003.07.011 14972206

[44] Lam SW Leenen LPH Van Solinge WW Hietbrink F Huisman A. Evaluation of hematological parameters on admission for the prediction of 7-day in-hospital mortality in a large trauma cohort. *Clin Chem Lab Med.* (2011) 49:493–9. 10.1515/CCLM.2011.069 21275817

[45] Lam SW Leenen LPH Van Solinge WW Hietbrink F Huisman A. Comparison between the prognostic value of the white blood cell differential count and morphological parameters of neutrophils and lymphocytes in severely injured patients for 7-day in-hospital mortality. *Biomarkers.* (2012) 17:642–7. 10.3109/1354750X.2012.712161 22998768

[46] Hesselink L Heeres M Paraschiakos F Ten Berg M Huisman A Hoefer IE A rise in neutrophil cell size precedes organ dysfunction after trauma. *Shock.* (2019) 51:439–46. 10.1097/SHK.0000000000001200 29889813

[47] Botha AJ Moore FA Moore EE Sauaia A Banerjee A Peterson VM. Early neutrophil sequestration after injury: a pathogenic mechanism for multiple organ failure. *J Trauma.* (1995) 39:411–7. 10.1097/00005373-199509000-00003 7473901

[48] van Hout GPJ van Solinge WW Gijsberts CM Teuben MPJ Leliefeld PHC Heeres M Elevated mean neutrophil volume represents altered neutrophil composition and reflects damage after myocardial infarction. *Basic Res Cardiol.* (2015) 110:58. 10.1007/S00395-015-0513-6 26467178 PMC4605987

[49] Köller M Wick M Muhr G. Decreased leukotriene release from neutrophils after severe trauma: role of immature cells. *Inflammation.* (2001) 25:53–9. 10.1023/A:1007027712387 11293666

[50] Pillay J Kamp VM Van Hoffen E Visser T Tak T Lammers JW A subset of neutrophils in human systemic inflammation inhibits T cell responses through Mac-1. *J Clin Invest.* (2012) 122:327–36. 10.1172/JCI57990 22156198 PMC3248287

[51] Chung HN Jong WC Lee J. Delta neutrophil index in automated immature granulocyte counts for assessing disease severity of patients with sepsis. *Ann Clin Lab Sci.* (2008) 38:241–6. 18715852

[52] Hellebrekers P Hesselink L Huisman A ten Berg M Koenderman L Leenen LPH Recognizing the mobilization of neutrophils with banded nuclei early after trauma. *Int J Lab Hematol.* (2020) 42:e224–7. 10.1111/ijlh.13272 32633074 PMC7586805

[53] Hampson P Dinsdale RJ Wearn CM Bamford AL Bishop JRB Hazeldine J Neutrophil dysfunction, immature granulocytes, and cell-free DNA are early biomarkers of sepsis in burn-injured patients: a prospective observational cohort study. *Ann Surg.* (2017) 265:1241–9. 10.1097/SLA.0000000000001807 27232244

[54] Tak T Wijten P Heeres M Pickkers P Scholten A Heck AJR Human CD62Ldim neutrophils identified as a separate subset by proteome profiling and in vivo pulse-chase labeling. *Blood.* (2017) 129:3476–85. 10.1182/blood-2016-07-727669 28515092

[55] Pillay J Den Braber I Vrisekoop N Kwast LM De Boer RJ Borghans JAM In vivo labeling with 2H2O reveals a human neutrophil lifespan of 5.4 days. *Blood.* (2010) 116:625–7. 10.1182/blood-2010-01-259028 20410504

[56] OverbeekeCTakTKoendermanL. The journey of neutropoiesis : how complex landscapes in bone marrow guide continuous neutrophil lineage determination. *Blood.* (2021) 39:2285–93. 10.1182/blood.2021012835 34986245

[57] Livingston DH Anjaria D Wu J Hauser CJ Chang V Deitch EA Bone marrow failure following severe injury in humans. *Ann Surg.* (2003) 238:748–53. 10.1097/01.sla.0000094441.38807.09 14578739 PMC1356155

[58] Cole E Davenport R Willett K Brohi K. The burden of infection in severely injured trauma patients and the relationship with admission shock severity. *J Trauma Acute Care Surg.* (2014) 76:730–5. 10.1097/TA.0b013e31829fdbd7 24487318

[59] Carp H. Mitochondrial N-formylmethionyl proteins as chemoattractants for neutrophils. *J Exp Med.* (1982) 155:264–75. 10.1084/JEM.155.1.264 6274994 PMC2186576

[60] Marasco WA Phan SH Krutzsch H Showell HJ Feltner DE Nairn R Purification and identification of formyl-methionyl-leucyl-phenylalanine as the major peptide neutrophil chemotactic factor produced by *Escherichia coli*. *J Biol Chem.* (1984) 259:5430–9. 10.1016/S0021-9258(18)91029-X 6371005

[61] Chatham WW Turkiewicz A Blackburn WD. Determinants of neutrophil HOCl generation: ligand-dependent responses and the role of surface adhesion. *J Leukoc Biol.* (1994) 56:654–60. 10.1002/JLB.56.5.654 7964173

[62] Ye RD Boulay F Ji MW Dahlgren C Gerard C Parmentier M International union of basic and clinical pharmacology. LXXIII. Nomenclature for the formyl peptide receptor (FPR) family. *Pharmacol Rev.* (2009) 61:119–61. 10.1124/PR.109.001578 19498085 PMC2745437

[63] Nauseef WM. How human neutrophils kill and degrade microbes: an integrated view. *Immunol Rev.* (2007) 219:88–102. 10.1111/J.1600-065X.2007.00550.X 17850484

[64] Li S Huang X Chen Z Zhong H Peng Q Deng Y Neutrophil CD64 expression as a biomarker in the early diagnosis of bacterial infection: a meta-analysis. *Int J Infect Dis.* (2013) 17:e902–13. 10.1016/j.ijid.2012.07.017 22940278

[65] Schiff DE Rae J Martin TR Davis BH Curnutte JT. Increased phagocyte FcγRI expression and improved Fcγ-receptor- mediated phagocytosis after in vivo recombinant human interferon-γ treatment of normal human subjects. *Blood.* (1997) 90:3187–94. 10.1182/blood.v90.8.3187 9376602

[66] Fjaertoft G Håkansson LD Pauksens K Sisask G Venge P. Neutrophil CD64 (FcgammaRI) expression is a specific marker of bacterial infection: a study on the kinetics and the impact of major surgery. *Scand J Infect Dis.* (2007) 39:525–35. 10.1080/00365540601113693 17577814

[67] Mahmoodpoor A Paknezhad S Shadvar K Hamishehkar H Movassaghpour AA Sanaie S Flow cytometry of CD64, HLA-DR, CD25, and TLRs for diagnosis and prognosis of sepsis in critically Ill Patients admitted to the intensive care unit: a review article. *Anesthesiol pain Med.* (2018) 8:e83128. 10.5812/AAPM.83128 30719416 PMC6347736

[68] Mahmoodpoor A Movassaghpour A Talebi M Shadvar K Soleimanpour H. Value of flow cytometry (HLA-DR, CD14, CD25, CD13, CD64) in prediction of prognosis in critically ill septic patients admitted to ICU: a pilot study. *J Clin Anesth.* (2020) 61:109646. 10.1016/J.JCLINANE.2019.109646 31708326

[69] Cong S Ma T Di X Tian C Zhao M Wang K. Diagnostic value of neutrophil CD64, procalcitonin, and interleukin-6 in sepsis: a meta-analysis. *BMC Infect Dis.* (2021) 21:384. 10.1186/s12879-021-06064-0 33902476 PMC8072745

[70] Thiriet C Mahjoub K Courte G Labroca P Cravoisy A Lemarie J Automated measurement of neutrophil CD64 expression for diagnosing sepsis in critically ill patients. *Minerva Anestesiol.* (2019) 85:943–50. 10.23736/S0375-9393.19.13420-7 30871305

[71] Ye Z Zou H Liu S Mei C Chang X Hu Z Diagnostic performance of neutrophil CD64 index in patients with sepsis in the intensive care unit. *J Int Med Res.* (2019) 47:4304–11. 10.1177/0300060519860677 31319721 PMC6753527

[72] Pfeifer R Pape HC. Diagnostik und versorgungsstrategien beim polytraumatisierten patienten. *Der Chir.* (2016) 87:165–75. 10.1007/s00104-015-0139-0 26830303

[73] Kolaczkowska E Kubes P. Neutrophil recruitment and function in health and inflammation. *Nat Rev Immunol.* (2013) 13:159–75. 10.1038/nri3399 23435331

[74] Bongers S Hellebrekers P Leenen LPH Koenderman L Hietbrink F. Intracellular penetration and effects of antibiotics on staphylococcus aureus inside human neutrophils: a comprehensive review. *Antibiotics.* (2019) 8:1–22. 10.3390/antibiotics8020054 31060222 PMC6628357

[75] Fraunholz M Sinha B. Intracellular *Staphylococcus aureus*: live-in and let die. *Front Cell Infect Microbiol.* (2012) 2:43. 10.3389/fcimb.2012.00043 22919634 PMC3417557

[76] Jämsä J Ala-Kokko T Huotari V Ohtonen P Savolainen ER Syrjälä H. Neutrophil CD64, C-reactive protein, and procalcitonin in the identification of sepsis in the ICU – post-test probabilities. *J Crit Care.* (2018) 43:139–42. 10.1016/J.JCRC.2017.08.038 28898742

